# Use of New Audio-Only Telemedicine Claim Modifiers

**DOI:** 10.1001/jamanetworkopen.2023.48224

**Published:** 2023-12-18

**Authors:** Anna M. Morenz, Jonathan Staloff, Joshua M. Liao, Edwin S. Wong

**Affiliations:** 1 Department of Medicine, University of Washington, Seattle; 2 Value and Systems Science Lab, University of Washington, Seattle; 3 Department of Family Medicine, University of Washington, Seattle; 4 Department of Health Systems and Population Health, University of Washington School of Public Health, Seattle

## Abstract

**Importance:**

Policymakers at both the state and federal levels face decisions about coverage of audio-only telemedicine amid a dearth of reliable data due to changes and variation in billing practices.

**Objective:**

To describe early trends in the use of new audio-only telemedicine claims modifiers 93 and FQ in Washington State, which were introduced to improve the designation and identification of audio-only telemedicine claims.

**Design, Setting, and Participants:**

This retrospective cohort study analyzed claims data from the Washington All-Payer Claims Database from January to November 2022. Participants included 4.3 million children and adults insured for at least 6 months in 2021 through public or private insurance plans.

**Exposures:**

Use of audio-only telemedicine was compared by age, race, ethnicity, insurance type, rurality, and Social Vulnerability Index.

**Main Outcomes and Measures:**

Audio-only telemedicine services were identified by claims appended by *Current Procedural Terminology* (*CPT*) code modifiers 93 or FQ or that included telephone-only *CPT* codes. Modifiers 93 and FQ denote audio-only telemedicine services for any reason and for behavioral health concerns, respectively.

**Results:**

In 2022, there were a total of 917 589 audio-only telemedicine services, of which 345 941 (38%) were appended with modifier FQ and 55 352 (6%) with modifier 93. Audio-only telemedicine services with these modifiers were most frequent for behavioral health diagnoses or routine prenatal and postpartum care. Individuals who used telemedicine exclusively via audio-only modality were more likely to be older (mean [SD] age, 46.0 [22.5] vs 42.0 [21.4] years) and insured by Medicare (41 758 of 196 225 [21%] vs 95 962 of 707 626 [14%]) than those who used at least 1 audiovisual service.

**Conclusions and Relevance:**

In this cohort study of a statewide all-payer claims database, modifiers 93 and FQ offered the important capability to identify audio-only telemedicine services beyond telephone-only *CPT* codes, but their uptake remained low. Audio-only telemedicine appears to offer an important means for access to behavioral health and perinatal care access, but further work is needed to study outcomes and quality of care.

## Key Points

**Question:**

How often were the new claims modifiers 93 and FQ, introduced to denote audio-only telemedicine services, used in Washington State in 2022?

**Findings:**

Based on this cohort study of 4.3 million children and adults with 17 589 audio-only telemedicine services from Washington state between January and November 2022, uptake of modifiers 93 and FQ was 6% and 38%, respectively. Modifiers were most frequently appended to claims for behavioral health diagnoses and routine prenatal and postpartum care.

**Meaning:**

In this study, uptake of new audio-only telemedicine claims modifiers 93 and FQ remained low; preliminary trends suggest that audio-only telemedicine may offer important means to access behavioral health and prenatal care.

## Teaser

This cohort study describes early trends in the use of new audio-only telemedicine claims modifiers 93 and FQ in Washington State, which were introduced to improve the designation and identification of audio-only telemedicine claims.

## Introduction

COVID-19 spurred a rapid uptake of telemedicine in the US. This adoption was enabled in part by responses to pandemic circumstances, including policy changes by Medicare and other insurers to reimburse for telemedicine services at parity to in-person services.^1^ Such changes included coverage and payment parity for audio-only telemedicine services that enabled patients and clinicians to connect synchronously via telephone.

However, audio-only telemedicine use has been difficult to characterize. At the beginning of the COVID-19 pandemic, audio-only telemedicine services were intended to be coded with specific *Current Procedural Terminology* (*CPT*) codes 99441 to 99443 (evaluation and management [E/M] telephone visits conducted with physicians) and 98966 to 98968 (telephone visits conducted with nonphysician health care professionals) in contrast to audiovisual services, which were denoted by *CPT* code modifiers 95 or GT.^2^ However, additional types of services denoted by other *CPT* codes were increasingly permitted to be offered via audio-only telemedicine over the course of the pandemic^3^ and difficult to capture due to a lack of specific modifiers.

Furthermore, among patients who self-reported having an audio-only service in a survey in fall 2020, only 20% had a matching corresponding claim.^4^ In comparison, among those reporting an audiovisual service, 96% had an audiovisual service claim. The reasons for this difference are unclear and may be due to discrepancies in billing (eg, clinician billing for audio-only services as audiovisual services) or perceptions (eg, differences in patients’ and clinicians’ definitions of audio-only services). Data discrepancies undercut the ability to accurately identify and understand utilization of audio-only telemedicine services.

This problem is particularly worrisome because of what is at stake. From a financial perspective, reimbursement for audio-only telemedicine is new, with ongoing debates about whether and how to continue reimbursement. This is, in part, due to the limited evidence regarding the clinical effectiveness of audio-only telemedicine and despite the lack of firmly established evidence, assumptions about the superiority of audiovisual telemedicine.^5,6^ In reality, some patients may prefer audio-only telemedicine when offered a choice.^7^

Audio-only telemedicine also poses important equity implications with historically disadvantaged groups potentially relying more heavily on audio-only modalities to access telemedicine. Specifically, use of audiovisual vs audio-only services has been lower among older, non-English speaking patients, racial and ethnic minority individuals, and individuals living in areas with low broadband access.^8,9^ This utilization gap may reflect access to, knowledge of, and comfort with the use of audiovisual-enabled devices, as well as practice and health care professional assumptions of who may be willing and able to conduct audiovisual services.

New *CPT* code modifiers 93 and FQ were created in 2022 to address these issues and more reliably identify situations in which audio-only medicine is used for any reason and specifically for behavioral health services, respectively. However, there is a dearth of data about the use of these codes, and the types of care they reflect. In this analysis, we aimed to address this knowledge gap and use new code modifiers to describe early trends in audio-only telemedicine.

## Methods

This study was reviewed and deemed not to be human participant research by the University of Washington institutional review board. This study conforms to the Strengthening the Reporting of Observational Studies in Epidemiology (STROBE) reporting guideline.^10^

### Audio-Only Telemedicine Services

Two audio-only telemedicine code modifiers were used in this analysis: 93 and FQ. Modifier 93 reflects any synchronous telemedicine services rendered via telephone or other real-time interactive audio-only telecommunications systems. Modifier FQ refers to a telemedicine behavioral health service furnished using audio-only communication technology. The modifier codes were developed by the *CPT* Editorial Panel within the American Medical Association. Both became effective January 1, 2022.^11^

### Setting and Data

As the largest purchaser of health care in the state, the Washington State Health Care Authority (HCA) administers Medicaid services for the state as well as public and school employees’ health insurance benefits. Beginning in 2022, HCA required clinicians to bill audio-only services using the appropriate modifier (93 or FQ).^11^ Commercial payers and Medicare also began to accept code modifiers during that year.

We conducted a retrospective cohort study utilizing data from the Washington State All-Payers Claims Database (WA-APCD) from calendar years 2021 and 2022. Eligibility data from 2021 were used for inclusion criteria, and claims data were analyzed from January to November 2022. WA-APCD includes data tracking individuals in 30 commercial health care payers, Medicaid managed care organizations, and Medicare Advantage plans.^12^ The WA-APCD does not include data from individuals enrolled in Medicare and Medicaid fee-for-service, most self-funded plans, and the Veterans Health Administration. Information stored in the WA-APCD includes all individual demographic characteristics and enrollment information, and all inpatient and outpatient claims reimbursed by payers. Claims include service procedure codes and modifiers that were used in constructing measures of audio-only telemedicine utilization.

Our overall cohort included individuals insured for at least 6 months in 2021 through public or private insurance plans. We excluded individuals from our cohort who were insured for less than 6 months of 2021. This exclusion sought to remove individuals residing briefly or transiently in Washington State. Analyses included individuals of all ages (children and adults).

### Variables

The primary outcome was a count of audio-only telemedicine services for each month from January to November 2022. Services were defined as audio-only for all claims appended with modifiers 93 or FQ as well as claims specifically corresponding to telephone visits with physicians or nonphysician health care professionals (with *CPT* codes 99441-99443 or 98966-98968, respectively). In addition, services were defined as audiovisual telemedicine for all *CPT* claims appended by modifiers 95 or GT.^13^

These definitions were used to create 2 patient groups defined based on telemedicine use. The first group (audio-only group) included individuals in the cohort who used telemedicine exclusively via audio-only services. The second group (audiovisual group) included individuals in the cohort who had at least 1 audiovisual telemedicine service with or without audio-only service use.

Characteristics used in analysis included age, sex, race, ethnicity, insurance type, rurality (based on Rural-Urban Commuting Area codes for individuals’ zip codes of residence^14^), and county-level Social Vulnerability Index (SVI).^15^ Race and ethnicity were based on the categories available in administrative claims data provided by insurers to the WA-APCD. We also examined the most common *International Statistical Classification of Diseases and Related Health Problems, Tenth Revision *(*ICD-10*) diagnostic codes and *CPT* codes accompanying the 93 and FQ modifiers.

### Statistical Analysis

We described baseline characteristics of the overall cohort as well as the audio-only and the audiovisual groups to compare characteristics of the 2 patient groups. We measured trends in utilization of audio-only telemedicine services over the 11-month period following implementation of new CPT code modifiers 93 and FQ from January to November 2022, stratified by code modifier.^11^ December 2022 was not included due to a lag in claims data availability. All analyses were conducted using R statistical software version 2022.01.1 (R Project for Statistical Computing).

## Results

### Cohort and Patient Group Characteristics

Out of the 4 669 536 unique individuals with insurance plan enrollment in 2021 identified in the WA-APCD, a total of 4 295 382 Washington State residents met inclusion criteria (individuals insured for at least 6 months in 2021 through public or private insurance plans) for the overall cohort. In this overall cohort, mean (SD) age was 37 (23) years, and 2 245 801 individuals (52%) were female (Table 1). For race, 2 296 600 (54%) had missing data, 81 255 (2%) were American Indian or Alaska Native, 116 602 (3%) were Asian, 175 256 (4%) were Black, 1 298 137 (30%) were White, and 287 953 (7%) were categorized as other (the WA-APCD does not maintain a record of race categories included in the other category). For ethnicity, 2 137 708 (50%) had missing data, 306 850 (7%) were Hispanic, and 1 850 824 (43%) were non-Hispanic. For insurance status, 1 948 277 (45%) had commercial insurance, 1 867 967 (44%) had Medicaid, and 479 138 (11%) had Medicare. For community characteristics, 522 325 (13%) resided in rural areas, and 468 547 (12%) lived in counties of the highest SVI quartile (highest vulnerability).

**Table 1.** Characteristics of Individuals in the Washington State All-Payer Claims Database Cohort Stratified by Telemedicine Use From January to November 2022

Characteristic	Patients, No. (%)		
	Overall cohort (N = 4 295 382)	Audio-only telemedicine (n = 196 225)	Any audiovisual telemedicine (n = 707 626)
Age, y			
Mean (SD)	37.2 (23.1)	46.0 (22.5)	42.0 (21.4)
<35	2 149 294 (50.0)	67 329 (34.3)	290 863 (41.1)
36-50	773 359 (18.1)	37 019 (18.9)	153 454 (21.7)
51-65	773 359 (18.0)	47 396 (24.2)	148 144 (20.9)
>65	595 162 (13.9)	44 481 (22.7)	115 195 (16.3)
Sex			
Female	2 245 801 (52.3)	116 391 (59.3)	439 554 (62.1)
Male	2 049 456 (47.7)	79 818 (40.7)	268 047 (37.9)
Race			
American Indian or Alaska Native	81 255 (1.9)	4737 (2.4)	9471 (1.3)
Asian	116 602 (2.7)	4566 (2.3)	13 897 (2.0)
Black	175 256 (4.1)	11 225 (5.7)	21 777 (3.1)
Native Hawaiian or Other Pacific Islander	39 579 (0.9)	1436 (0.7)	3283 (0.5)
White	1 298 137 (30.2)	57 478 (29.3)	213 186 (30.1)
Other^a^	287 953 (6.7)	12 117 (6.2)	25 677 (3.6)
Missing	2 296 600 (53.5)	104 666 (53.3)	420 335 (59.4)
Ethnicity			
Hispanic	306 850 (7.1)	13 188 (6.7)	23 298 (3.3)
Non-Hispanic	1 850 824 (43.1)	81 029 (41.3)	286 354 (40.5)
Missing	2 137 708 (49.8)	102 008 (52.0)	397 974 (56.2)
Insurance type			
Commercial	1 948 277 (45.4)	64 804 (33.0)	365 358 (51.6)
Medicaid	1 867 967 (43.5)	89 663 (45.7)	246 306 (34.8)
Medicare	479 138 (11.2)	41 758 (21.3)	95 962 (13.6)
Rural	522 325 (12.9)	17 113 (8.8)	64 022 (9.5)
SVI quartile			
Low (0-0.25)	226 674 (5.6)	8752 (4.5)	37 157 (5.5)
Low to medium (0.26-0.50)	2 186 757 (54.0)	115 037 (59.3)	412 234 (61.2)
Medium to high (0.51-0.75)	1 166 735 (28.8)	53 750 (27.7)	174 191 (25.9)
High (0.76-1.0)	468 547 (11.6)	16 561 (8.5)	50 075 (7.4)

^a^ The Washington State All-Payers Claim Database does not maintain a record of race categories included in the other category.

There were 196 225 individuals (5% of the overall cohort and 22% of the subpopulation who had any telemedicine service claims) in the audio-only patient group. The mean (SD) age of 46 (23) years, and 116 391 individuals (59%) were female (Table 1). The distribution of race and ethnicity was similar to the overall cohort. For insurance status, 64 804 (33%) had commercial insurance, 89 663 (46%) had Medicaid, and 41 758 (21%) had Medicare. For community characteristics, 17 133 (9%) resided in rural areas, and 16 561 (9%) in the highest SVI quartile.

There were 707 626 individuals (16% of the overall cohort and 78% of the population who had any telemedicine service claims) in the audiovisual patient group. Among individuals in this group, the mean (SD) age was 42 (21) years and 439 554 individuals (62%) were female (Table 1). Overall, 23 289 (3%) were Hispanic, and 286 354 (41%) were non-Hispanic, with race and ethnicity data missing for many individuals (race, 60%; ethnicity, 56%). For insurance status, 365 358 (52%) had commercial insurance, 246 306 (35%) had Medicaid, and 95 962 (14%) had Medicare. For community characteristics, 64 022 (9%) resided in rural areas, and 50 075 (8%) in the highest SVI quartile.

### Audio-Only Telemedicine Trends

In 2022, the overall cohort of nearly 4.3 million individuals generated a total of 917 589 audio-only telemedicine services, of which 345 941 (38%) were appended with modifier FQ and 55 352 (6%) with modifier 93 for a total penetration of 44%. As shown in the Figure, monthly audio-only telemedicine claims were 92 228 in January 2022, oscillated until a second, lower peak in August 2022 at 88 388, and then declined to a nadir of 74 011 in November 2022. In January 2022, claims with the FQ modifier accounted for 22 375 services (24% of all audio-only telemedicine claims in January 2022), peaked at 47 865 claims (53%) in June 2022, and then declined back to 22 475 claims (30%) in November 2022. Claims with the 93 modifier were low throughout 2022, but increased from 362 claims in January 2022 (0.4%) to 8356 claims in November 2022 (11%). A total of 309 020 claims were captured with the 93 and FQ modifiers that would not have been captured with audio-only *CPT* codes alone (99441-99443 and 98966-98968). In comparison, the overall cohort generated a total of 3 335 769 claims for audiovisual telemedicine services (denoted by modifiers 95 or GT), which slowly declined over the course of 2022 from 400 669 claims in January 2022 to 260 244 in November 2022 (eFigure in Supplement 1).

**Figure.**
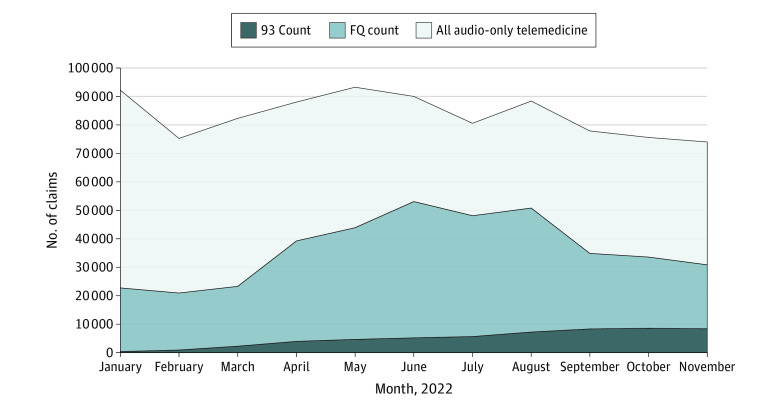
Trends in Audio-Only Telemedicine Code Modifier Use in 2022

### Characteristics of Modifier 93 and FQ Use

We identified a total of 55 352 claims using modifier 93 (Table 2). The 5 most common diagnostic codes associated with claims using modifier 93 were anxiety disorder (4035 audio-only telemedicine claims [7%]), posttraumatic stress disorder (PTSD; 3803 [7%]), encounters for routine prenatal care (3546 [6%]) or postpartum follow-up (3444 [6%]), and major depressive disorder (2238 [4%]). The most common *CPT* codes associated with modifier 93 were telephone E/M codes (16 489 [30%]), psychotherapy codes (12 199 [22%]), E/M of an established patient (9428 [17%]), registered nurse services (3544 [6%]), and family training and counseling for child development (2316 [4%]).

**Table 2.** Characteristics of Audio-Only Telemedicine Claims Using Modifiers 93 and FQ in the Washington State All-Payer Claims Database From January to November 2022

Characteristic	Modifier 93 (n = 55 352)	Modifier FQ (n = 345 941)
Unique individuals who received claims, No.	27 011	113 093
Ratio of claims to individuals	2.05	3.06
Most common diagnostic codes (No. [%] of total claims)	Anxiety disorders (4035 [7.3]) Posttraumatic stress disorder (3803 [6.9]) Encounter for routine prenatal care (in first, second, or third trimesters) (3546 [6.4]) Encounter for routine postpartum follow-up (3444 [6.2]) Major depressive disorder (2238 [4.0])	Major depressive disorder (54 284 [15.7]) Posttraumatic stress disorder (39 603 [11.4]) Anxiety disorders (35 793 [10.3]) Schizophrenia or schizoaffective disorders (15 064 [4.4]) Bipolar disorders (12 773 [3.7])
Most common *CPT* codes (No. [%] of total claims)	Telephone E/M codes 99441-3 (16 489 [29.8]) Psychotherapy codes 90832, 90834, 90837 (12 199 [22.0]) E/M of an established patient 99211-5 (9428 [17.0]) RN services, up to 15 min (3544 [6.4]) Family training and counseling for child development, per 15 min (2316 [4.2])	Psychotherapy codes 90832, 90834, 90837 (88 573 [25.6]) Telephone E/M codes 99441-3 (73 595 [21.3]) Behavioral health counseling and therapy, per 15 min (39 970 [11.6]) E/M of an established patient 99211-5 (36 878 [10.7]) Comprehensive community support services, per 15 min (26 425 [7.6])

Abbreviations: *CPT*, *Current Procedural Terminology*; E/M, evaluation and management; RN, registered nurse.

There were 345 941 claims using modifier FQ. The 5 most common diagnostic codes associated with modifier FQ were major depressive disorder (54 284 [16%]), PTSD (39 603 [11%]), anxiety disorders (35 793 [10%]), schizophrenia or schizoaffective disorders (15 064 [4%]), and bipolar disorders (12 773 [4%]). The most common *CPT* codes associated with modifier FQ were psychotherapy codes (88 573 [26%]), telephone E/M codes (73 595 [21%]), behavioral health counseling and therapy (39 970 [12%]), E/M of an established patient (36 878 [11%]), and comprehensive community support services (26 425 [11%]).

## Discussion

Early analysis from Washington State revealed the role of new audio-only telemedicine *CPT* modifiers in identifying services that would not have been identified by telephone visit *CPT* codes alone. Claims with new modifiers represented 25% of all audio-only telemedicine services in January 2022 and increased to a peak of 60% in July 2022, with overall penetration of 44%.

Modifiers 93 and FQ represent a concerted effort to improve the identification of audio-only telemedicine services within claims data beyond the traditional *CPT *codes exclusively used for audio-only services (99441-99443 and 98966-98968), as the potential service types for which audio-only telemedicine can be used has expanded since 2020. This is a needed first step to more accurately describe utilization patterns, quality, and equity of audio-only telemedicine and to inform policy decisions moving forward beyond the public health emergency. Potential reasons that uptake of these new modifiers by clinicians were not even greater than observed include lack of familiarity and lack of specific guidance from Medicare and commercial payers on their use. Nonetheless, given increasing interest from payers, including Medicare as of January 1, 2023,^16^ in requiring clinicians to utilize these modifiers, the 93 and FQ modifiers will likely continue to evolve as a valuable means to reliably identify audio-only telemedicine claims to conduct the research on quality, outcomes, and equity needed to inform policy.

Consistent with prior work on disparities in audio-only telemedicine use,^7,8,9^ those who used telemedicine exclusively via audio-only were older (46 vs 42 years) and more likely to be insured by Medicare (21% vs 14%). Individuals who used telemedicine exclusively via audio-only were also more likely to be insured by Medicaid and less likely to be commercially insured than those who used an audiovisual service at least once. Those who used any telemedicine (exclusively audio-only or at least 1 audiovisual service) were less likely to be rural residents and less likely to reside in the highest quartile of SVI than the overall cohort, potential inequities that merit further investigation.

Audio-only telemedicine claims with modifiers 93 and FQ were frequently utilized for common behavioral health diagnoses, including anxiety, depression, PTSD, schizophrenia, and bipolar disorders, signaling the importance of audio-only telemedicine for facilitating the close and frequent follow-up often required for behavioral health issues. In line with the frequent behavioral health diagnostic codes, the most common *CPT* codes used were psychotherapy or behavioral or family counseling, in addition to telephone E/M and standard E/M for an established patient. Early uptake of modifiers 93 and FQ has been high among those providing care to individuals with behavioral health needs, which may reflect the fact that Medicaid required use of these modifiers in Washington State in 2022 and is the largest payer for behavioral health services in the United States.^17^ However, behavioral health may lend itself particularly well to audio-only telemedicine services given that a physical examination is often unnecessary, especially if a clinician has been able to establish care in person initially with a patient.

Routine prenatal and postpartum care were also common diagnoses used with modifiers 93 (compromising 13% of all claims with modifier 93). Indeed, like many areas of care, prenatal care saw a rapid and novel uptake of telemedicine during the pandemic.^18,19^ Given the overall low count of services with modifier 93, further work would be required to specifically describe trends in telemedicine use for routine prenatal and postpartum care and the impact that this care access may have on maternal and infant outcomes, which are of great importance given the marked racial disparities in the US in maternal health outcomes including mortality.^20^ Additionally, both perinatal care and behavioral health care are important areas of health care for access to be facilitated given inequities in health outcomes in these areas at least partially driven by access to care.^20,21^

### Limitations

This study has limitations, including high levels of missing data in the WA-APCD for race and ethnicity. Also, data do not include additional variables that could help explain audio-telemedicine use, such as broadband access or primary language. Because WA-APCD does not include data from certain insurance plans, such as Medicare and Medicaid fee-for-service and the Veterans Health Administration, utilization trends may not generalize to all individuals. Furthermore, interpretation of the uptake of modifiers 93 and FQ is limited by the inability to identify the true denominator of performed audio services. Nonetheless, this study provides early insights into the patterns of use of audio-only telemedicine modifiers 93 and FQ from more than 30 insurers in Washington State, one of the first states in which a major public insurer required use of the 93 and FQ modifiers.

## Conclusions

Early trends in use of modifiers 93 and FQ in Washington State reveal penetrance of 44% into overall audio-only telemedicine claims by the end of the year in 2022, with increases to be anticipated as Medicare and other commercial payers require their use. Overall, audio-only telemedicine utilization remains lower than audiovisual service utilization, with 917 589 claims compared with 3.3 million, respectively, although audio-only telemedicine monthly service counts were more stable over the course of 2022 compared with a steady decline in monthly audiovisual service counts. Further research should assess the clinical quality of audio-only telemedicine services compared with other alternatives and address the differences in audio-only vs audiovisual telemedicine use noted early in the pandemic, which appear to persist. Intermediate-term policies should also aim to ensure that equitable access to telemedicine remains in place while efforts to improve systemic and affordable broadband access and match modality to patient preferences move forward.

## Notes

Supplement 1. **eFigure.** Trends in Audiovisual Telemedicine Use in 2022

Supplement 2.
Data Sharing Statement
