# Supplementary material for: Use of New Audio-Only Telemedicine Claim Modifiers
Source: JAMA Netw Open. 2023 Dec 18;6(12):e2348224. doi: 10.1001/jamanetworkopen.2023.48224 (PMC10728765; doi:10.1001/jamanetworkopen.2023.48224)
Supplement: Supplement 1. — eFigure. Trends in Audiovisual Telemedicine Use in 2022 [file jamanetwopen-e2348224-s001.pdf]

Supplemental Online Content

Morenz AM, Staloff J, Liao JM, Wong ES. Use of new audio-only telemedicine claim modifiers. *JAMA Netw Open.* 2023;6(12):e2348224. doi:10.1001/jamanetworkopen.2023.48224

eFigure. Trends in Audiovisual Telemedicine Use in 2022

This supplemental material has been provided by the authors to give readers additional information about their work.

© 2023 Morenz AM et al. *JAMA Network Open.*

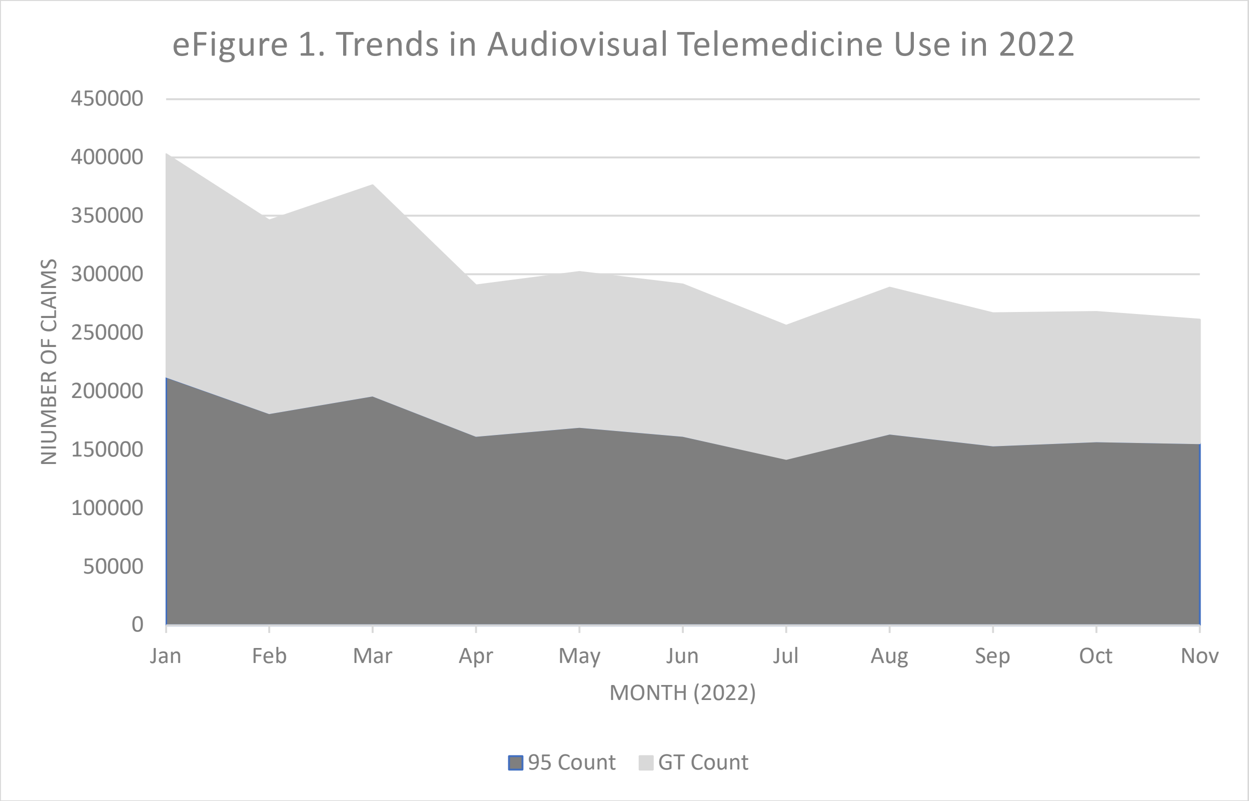

95 and GT- Modifiers that Indicate Audiovisual Telemedicine Services

© 2023 Morenz AM et al. *JAMA Network Open.*
