# Supplementary material for: Use of New Audio-Only Telemedicine Claim Modifiers
Source: JAMA Netw Open. 2023 Dec 18;6(12):e2348224. doi: 10.1001/jamanetworkopen.2023.48224 (PMC10728765; doi:10.1001/jamanetworkopen.2023.48224)
Supplement: Supplement 2. — Data Sharing Statement [file jamanetwopen-e2348224-s002.pdf]

Data Sharing Statement

Morenz. Use of New Audio-Only Telemedicine Claim Modifiers. *JAMA Netw Open*. Published December 18, 2023. doi:10.1001/jamanetworkopen.2023.48224

Data
Data available: No

Additional Information
Explanation for why data not available: Data is accessed in an all payer claims database virtual resource enclave, which precludes removal of any data.
